# An analysis of the nutritional effects of *Schisandra chinensis* components based on mass spectrometry technology

**DOI:** 10.3389/fnut.2023.1227027

**Published:** 2023-07-25

**Authors:** Mengzhen Jia, Li Zhou, Yuanyuan Lou, Xiaoqing Yang, Hangyu Zhao, Xinshou Ouyang, Yanjie Huang

**Affiliations:** ^1^Department of Pediatrics, Henan University of Chinese Medicine, Zhengzhou, Henan, China; ^2^School of Pharmacy, China Pharmaceutical University, Nanjing, Jiangsu, China; ^3^Department of Pediatrics, The First Affiliated Hospital of Henan University of CM, Zhengzhou, Henan, China; ^4^Department of Internal Medicine, Digestive Disease Section, Yale University, New Haven, CT, United States

**Keywords:** *Schisandra chinensis* (Turcz.) Baill, *Schisandra sphenanthera* Rehd. et Wits, mass spectrometry, active components, nutritional value, dietary supplements

## Abstract

**Objective:**

*Schisandra chinensis* (Turcz.) Baill. (*S. chinensis*) is a Traditional Chinese medicinal herb that can be used both for medicinal purposes and as a food ingredient due to its beneficial properties, and it is enriched with a wide of natural plant nutrients, including flavonoids, phenolic acids, anthocyanins, lignans, triterpenes, organic acids, and sugars. At present, there is lack of comprehensive study or systemic characterization of nutritional and active ingredients of *S. chinensis* using innovative mass spectrometry techniques.

**Methods:**

The comprehensive review was conducted by searching the PubMed databases for relevant literature of various mass spectrometry techniques employed in the analysis of nutritional components in *S. chinensis*, as well as their main nutritional effects. The literature search covered the past 5 years until March 15, 2023.

**Results:**

The potential nutritional effects of *S. chinensis* are discussed, including its ability to enhance immunity, function as an antioxidant, anti-allergen, antidepressant, and anti-anxiety agent, as well as its ability to act as a sedative-hypnotic and improve memory, cognitive function, and metabolic imbalances. Meanwhile, the use of advanced mass spectrometry detection technologies have the potential to enable the discovery of new nutritional components of *S. chinensis*, and to verify the effects of different extraction methods on these components. The contents of anthocyanins, lignans, organic acids, and polysaccharides, the main nutritional components in *S. chinensis*, are also closely associated to its quality.

**Conclusion:**

This review will provide guidelines for an in-depth study on the nutritional value of *S. chinensis* and for the development of healthy food products with effective components.

## 1. Introduction

In recent years, with the rapid development of the Traditional Chinese Medicine industry, people are paying more attention tos their health, and many health food products, especially Chinese herbal medicines with dual functions as medicine and food, have become increasingly popular. *Schisandra* is a medicinal and edible plant. *Schisandra* can be classified predominantly into *Schisandra chinensis* (Turcz.) Baill. (*S. chinensis*) and *Schisandra sphenanthera* Rehd. et Wils (*S. sphenanthera*). In accordance to the *Compendium of Materia Medica*, *S. chinensis* has higher medicinal value than *S. sphenanthera* (1). *S. chinensis* is a deciduous vine plant in the Magnoliaceae family, also known as *Kadsura chinensis, Maximoviczia amurensis, Maximoviczia chinensis, Omija, Maximoviczia japonica, Speerostemma japonicum*, Wuweizi (2), characterized by five tastes: sweet, bitter, pungent, salty, and sour (3). The species of natural *S. chinensis* grows in mixed forests and shrubs in the Northeast and North China, North Korea, and the Far East of Russia. Owing to its effects of astringing and securing, tonifying qi and engendering fluid, as well as tonifying the kidney and calming the heart (4). *S. chinensis* has been frequently used as a tonic Chinese medicine in clinical practice. WuWeiZiJiangNang, which is processed from the mature fruit of *S. chinensis*, is also a dietary supplement. Since 2007, this plant has been listed as an valuable medicinal plant in the International Pharmacopeia published by the World Health Organization (5). *S. chinensis* contains a variety of beneficial nutrients such as lignans, phenolic acids, flavonoids, triterpenoids, organic acids, vitamins, and polysaccharides. In addition to being widely used as an effective additive in medicine, cosmetics, and health products, it is suitable for use in tea, beverages, jam, and seasonings as a functional food ingredient with unique flavor (3, 6, 7). In recent years, research on the components and functions of *S. chinensis* was numerous and jumbled, with mainly antioxidant, antibacterial, anti-inflammatory, anti-aging and anticancer, as well as the prevention of chronic diseases related to diet and other effects attributed to *S. chinensis* extracts and its single constituent (8–11). In Russia, *S. chinensis*, as a plant adaptation agent, has been found to be useful in strengthening the body’s resistance to stress and offers stress-protective effects against various harmful factors such as hot and cold stimuli (12). The *S. chinensis* extracts serve as a good natural preservative, inhibiting the activity of *Escherichia coli*, destroying the cell membrane and wall of *Staphylococcus aureus* (13), and inhibiting *Listeria monocytogenes*, *Clostridium perfringens* and *Salmonella* (14).

In recent years, advances in chromatography-mass spectrometry (MS) analysis technology has greatly contributed to the rapid, high-throughput qualitative and accurate quantification of complex components in Chinese medicinals (15). Recently, High-resolution MS (HRMS) has been used to discover new bioactive substances such as nutrients in herbal products and dietary supplements, to identify components of food pollutants, and to investigate the pharmacokinetics, oral bioavailability, and tissue distribution of effective ingredients in Chinese medicinals (15, 16). Considering the advantages of fast analysis speed, easy operation, and low organic solvent consumption, the direct analysis in real time ionization source coupled with quadrupole orbitrap MS (DART-Q-Orbitrap MS) has been applied to rapidly detecting and identifying the components of *S. chinensis* and *S. sphenanthera*, and identifying the differences between the two components at the MS level (17). In addition, the extractive nanoelectrospray ionization-mass spectrometer (EnESI-MS) with high sensitivity and specificity was formed by coupling the extractive nanoelectrospray ionization source and a high resolution mass spectrometer, which has been used to authenticate *S. chinensis* and *S. sphenanthera* with similar shape and different efficacy (18).

The above MS techniques have been mainly applied to identifying the active components of *S. chinensis* for its medicinal potential. Currently, there is a lack of comprehensive study or systemic characterization of the nutritional and active components of *S. chinensis* by using innovative MS techniques. Initial search in PubMed database was made. Using “*Schisandra chinensis* (Turcz.) Baill.,” “*S. chinensis*,” “Wuweizi,” “*Kadsura chinensis*,” “*Maximoviczia amurensis*,” “*Maximoviczia chinensis*,” “*Omija*,” “*Maximoviczia japonica*” and “*Speerostemma japonicum*,” and “mass spectrometry” as search terms. The search was combined with a screening for literature in reference sections of relevant studies. Following search criteria “mass spectrometry technology related to components analysis of *S. chinensis*” and “nutritional effects,” a total of 84 studies were included in the review, and these studies were published predominantly during the past 5 years. Various bioactive and nutritional components of *S. chinensis* detected by MS are systematically summarized in order to facilitate the discovery of new extraction methods and components of *S. chinensis.* Moreover, the nutritional and health functions and mechanisms of the active ingredients associated with *S. chinensis* are also evaluated. The aim of this review is to provide theoretical guidelines for the prevention and health care, as well as the therapeutic value of *S. chinensis*.

## 2. Nutritional and active components of *Schisandra chinensis*

### 2.1. Polyphenols

Polyphenols, also known as the “the seventh category of nutrients,” have avariety of functions including antioxidant, anti-aging, antiviral, lowering blood sugar and lipids, protecting the heart and nerves. Flavonoids and phenolic acids, which belong to the polyphenols, as secondary metabolites of plants and widely present in fruits, flowers, seeds, leaves, roots, or other parts of plants, and they are associated with antioxidant properties (19). The leaves of *S. chinensis* are predominantly flavonoids and phenolic acids (20). High-performance liquid chromatography-diode array detection (HPLC-DAD) was utilized to analyze and compare the type and content of polyphenolic compounds in microshoot cultures of *S. chinensis* cultivated on agar medium and in the fruits and seeds of raw *S. chinensis* (21). The results showed that there were 8 types of phenolic acids present in the *vitro* culture, namely chlorogenic, cryptochlorogenic, gallic, neochromogenic, protocatalytic, salicylic, syringic and vanillic acids. Among these 8 of phenolic acids, neochromogenic and chlorogenic acids had the higher contents, as well as two types of flavonoids: kaempferol and quercitrin, with quercetin having the highest contents (21). The highest dry weight of total phenolic acids and flavonoids in *S. chinensis* micro bud cultures were 357.93 mg/100 g and 105.07 mg/100 g, respectively. These contents were 1.59 times and 5.95 times lower than the parental plant leaf extracts, and 4.30 times and 1.25 times higher than the fruit extracts. This study not only indicated that the contents of phenolic acids and flavonoids in leaves were higher than those in fruits, but also provided an optimized protocol for *vitro* cultivation of *S. chinensis* (21).

A study evaluated the polyphenol content of the fruits and leaves of *S. chinensis* using HPLC-ultraviolet spectroscopy-MS (HPLC-UV-MS) (22). The results showed that the main composition of flavonoid in the leaves was isoquercetin, followed by quercetin, while the main flavonoid in the fruits was rutin, but its contents were lower than that in leaves, which was consistent with the significantly higher antioxidant activity of leaf extracts compared to fruits (22). Quercetin is commonly used as a dietary ingredient and supplement in the daily diet, exerting the function of a functional food (23). Enzymatically modified isoquercetin (EMIQ) is an isoquercitrin derivative obtained from rutin through enzymatic transformations, which improves the bioavailability of isoquercitrin (24). Furthermore, EMIG has higher safety and biological activity, as well as various healthful biological characteristics such as antiallergic, anti-inflammatory, protecting the heart and nerves, and regulating lipid metabolism (24). EMIQ has attracted increasing attention from researchers who are working on making it as a food additive and as an ingredient in dietary supplements (24). A recent study has found that quercetin and rutin exhibit good radiation stability, and that exposure to ionizing radiation does not alter their chemical structure and antioxidant properties (25). In a double-blind clinical study, rutin has been shown to increase skin elasticity and reduce wrinkles length, area, and number when applied to the skin of subjects aged 30–50 (26). This anti-aging function may be achieved by increasing mRNA expression of collagen type I α1 and reducing mRNA expression of matrix metallopeptidase 1 in human dermal fibroblasts. In addition, it may also promote the clearance of aging-related reactive oxygen species (ROS) in a dose-dependent manner (26). Anti-hyperglycaemia effects of rutin include reducing the absorption of carbohydrates in the small intestine, inhibiting tissue gluconeogenesis, increasing tissue glucose uptake, stimulating β Cells to secrete insulin, and protecting the Langerhans islet from degeneration (27). Additionally, Rutin can reduce sorbitol, ROS, advanced glycation end-product precursors, and inflammatory cytokines, thereby preventing or treating diabetes and its complications (27). Furthermore, a number of neurodegenerative diseases have been shown to benefit from rutin, including Alzheimer’s disease, Parkinson’s disease, Huntington’s disease and prion disease, which caused by neuron loss, apoptosis, mitochondrial dysfunction, oxidative stress, and inflammation (28). It has been shown that rutin is effective in treating cancer at varying degrees by regulating cellular signaling pathways, such as Wnt/β-catenin, p53 independent pathway, PI3K/Akt, JAK/STAT, mitogen-activated protein kinase (MAPK), p53, and NF-ĸB pathway (29). Several studies have indicated that the alcohol extract and aqueous extract of *S. chinensis* have distinct components. Six phenolic acids were detected in the methanol extracts, including chlorogenic, p-coumaric, p-hydroxybenzoic, protocatechuic, salicylic, and syringic acids, while namely p-coumaric and syringic acid were the highest in *S. chinensis* extracts (30). The aqueous extracts of *S. chinensis* were analyzed using HPLC–MS. In addition to lignans, phenolic acids and flavonoids, as well as protocatechiuc and p-coumaril quinic acids were found to be predominant (31). There are two major categories of flavonoids identified: Quercetin derivatives, including glucosides, galactosides, and rutinosides and Kaempferol derivatives, including rutinoside and glucoside (31).

*S. chinensis* bee pollen is also an important functional food with a long history of sales in China. Cheng et al. (32) detected the total phenolic acids quantified were 36.11 mg/kg, and the total flavonoids quantified were 1643.31 mg/kg in *S. chinensis* pollen extracts by HPLC with DAD and electrochemical detector. The most abundant phenolic compound was quercetin (719.93 mg/kg), followed by galangin, hesperetin, and resveratrol. It is noteworthy that a species in the genus *Schisandra* is generally called *Schisandra rubriflora* (Franch.) Rehd. et Wils (*S. rubriflora*), native to Western Sichuan province of China, which is less studied and used (33). Recently, Szopa et al. found that *S. rubriflora* contains abundant phenolic substances (34). Researchers identified 27 phenolic compounds from the *S. rubriflora* fruit, stem, leaf and *in vitro* micro stem culture extracts by using ultra-high-performance liquid chromatography with a photodiode array detector coupled to electrospray ionization ion trap MS (UHPLC-DAD-ESI-MS^3^), and demonstrated that for the first time there was close correlation between the total phenolic content of *S. rubriflora* and antioxidant potential (34). A flavonoid compound known as anthocyanins is an important component of the fruit pigment, playing a vital role in determining the coloration of the fruits (35). An optimal extraction condition could yield 29.6 mg/g of anthocyanins from *S. chinensis* fruit (36). The anthocyanins present in *S. chinensis* have been characterized using HPLC-ESI-MS. Upon acid hydrolysis, the purified Cya-3-O-xylrut, the mostly colorant of *S. chinensis*, was successfully purified. This compound from the water extract of *S. chinensis* accounted for more than 86% of total antioxidant activity (37). A HPLC-ESI-MS was utilized to determine the contents of four major anthocyanins, CyXylGlu, CyGluRutin, CyRutin and CyXylRutin in *S. chinensis* fruits, with CyXylRutin (cyanidin3-*O*-xyl-rutinoside) being the most abundant (35, 38). In *S. chinensis* fruits, CyXylRutin is the key anthocyanin in determining the reddening appearance. The genetic components involved in the biosynthesis of cyanidin3-*O*-xyl-rutinoside have been identified by combining SMRT sequencing with second-generation sequencing and targeted metabolomics analysis (38). Meanwhile, the complete anthocyanin biosynthesis pathway in *S. chinensis* was constructed, and five *ScMYBs*, three *ScbHLHs*, and two *ScWD40s* were identified as being involved in this process, which may function in anthocyanin synthesis (38). Based on these findings, further investigation into the molecular mechanism and the gentic regulation of anthocyanin biosynthesis can be carried out in order to improve the appearance and quality of *S. chinensis* fruit. The cyanidin 3-Rutinoside, one of the most abundant components in fruits and vegetables, was detected in *S. chinensis* using LC-ESI-triple quadrupole-MS. The study has demonstrated that cyanidin 3-Rutinoside can inhibit the secretion of inflammatory cytokines such as IL-6 and tumor necrosis factor-α (TNF-α) along with NF-ĸB phosphorylation. This, in turn, can improve allergic inflammation induced by PMA/A23187 in human mast cell line (39). Therefore, cyanidin 3-Rutinoside may be useful as a therapeutic agent in the treatment of allergic diseases. In addition, UHPLC-quadrupole time of flight-MS (UHPLC-Q-TOF-MS), was used to determine the chemical structures of proanthocyanidins (which can be decomposed into anthocyanins) and identified 12 substances in *S. chinensis* seed coat, including Malic acid, Citric acid, (Epi) gallocatechin, Protocatechuic acid, Procyanidin trimer mixed, Procyanidin B4, Procyanidin trimer 1, Catechin, Procyanidin tetramer, Procyanidin trimer 2, Procyanidin trimer 3, Procyanidin dimer 2, and 1 with unspecified substance (40). Despite this, the function of these new active ingredients have yet to be discovered.

### 2.2. Lignins

#### 2.2.1. Types and functions of lignans in *Schisandra chinensis*

Lignans are a class of natural compounds derived from two or more phenylpropanoid units (a structure of C_6_-C_3_) (41). Up to date, more than 170 lignans have been identified from *S. chinensis* and *S. sphenanthera*, which can be divided into six groups: dibenzocyclooctadiene ligans, tetrahydrofuran ligans, dibenzylbutane ligans, aryltetralin ligans, dihydrobenzofuran ligans, and furofuran ligans. Since Dibenzocyclooctadiene lignans are the predominant bioactive constituents of *S. chinensis* fruits, they are often referred to as “Schisandra lignans” in the scientific literature (42). It is noteworthy that during the research process, there have been multiple synonymous names assigned to the active components of *S. chinensis* lignans. The mixed use of these names can lead to confusion for scientists to understand and comprehend the active components of *S. chinensis*. The article provided supplement in organizing the synonymous names of *S. chinensis* lignans in Table 1. Lignans may exist in variety of plant parts, such as seeds, stems, leaves, and roots in both free and glycosidic forms (43). The *S. chinensis* fruit contains the highest amount of lignans, accounting for approximately 2% of its dry weight (44). A comparative study was conducted on the amount of lignan in *S. chinensis* extracts of fruit, leaves, stems, and roots using ultra performance liquid chromatography (UPLC)-Q-TOF-MS (45). The researchers found that the roots contained higher contents of gomisin D, schisandrol B, schisanterin C, kadsuranin, and kadlongilactone F than those in the fruits, and these components were closely related to their antioxidant and anti-inflammatory activities (45). The antioxidant capacity of the main lignans in *S. chinensis* extract was determined by using the HPLC-online TEAC method. The result shown that gomisin D was the only lignan that could scavenge ABTS^+^ radicals (46).

**Table 1:** The lignans and their synonymous names (42).

Lignans	Synonymous names
schisandrin	schisandrol A, schizandrin, wuweizichun A, wuweizi alcohol A
schisandrin A	schizandrin A, deoxyschisandrin, deoxyschizandrin, dimethylgomisin J, wuweizisu A
schisandrol B	gomisin A, besigomsin, wuweizi alcohol B, wuweizichun B
schisandrin B	Schizandrin B, Wuweizisu B, Gomisin N, γ-Schisandrin, Isokadsuranin, Deoxygomisin A
Schisantherin A	Schizantherin A, Gomisin C, schisandrer A, Wuweizi ester A
Schisantherin B	Schizantherin B, Gomisin B, Schisandrer B, Wuweizi ester B
Schisandrin C	Schizandrin C, Wuweizisu C
Gomisin M1	Gomisin L1
Schisanhenol	Gomisin K3

Schisandrin A, B and C, schisandrol B, schisantherin A-B were the main components in dibenzocyclooctadiene lignans, and there were significant differences in the amount of these components between *S. chinensis* and *S. sphenanthera* (42). The researchers cultured *S. chinensis* in a liquid medium system, and quantitatively analyzed the content of *S. chinensis* lignans using HPLC-DAD and LC-DAD-ESI-MS (4). Results showed that four major lignans were detected, namely schisandrin (syn. Schisandrol A), angeloyl−/tigloylgomisin Q, deoxyschisandrin (syn. Schisandrin A) and gomisin A (syn. Schisandrol B), and their maximum dry weights were 65.62 mg/100 g, 49.73 mg/100 g, 43.65 mg/100 g, and 34.36 mg/100 g, respectively (4). A study conducted using supercritical fluid chromatography and DAD detected the presence of 9 lignans in *S. chinensis,* and the results indicated that schisandrol A was the most abundant lignan, followed by schisandrin B (syn. Gomisin N) or schisandrol B (47). Therefore, Schisandrol A is the predominant component of lignans in *S. chinensis*. A study showed that schisandrol A has an active antidepressant effect on lipopolysaccharide induced depression in mice by regulating intestinal microbiota and inhibiting TLR4/NF-κB signal pathways in the hippocampus to reduce neuroinflammation (48). A study *in vitro* and *vivo* showed that schisandrol A inhibits pulmonary fibrosis by regulating TGF- β signal pathway (49). Schizandrol A promoted the activation of PI3K/Akt in acute myocardial ischemia mice and H9c2 cells treated with oxygen–glucose deprivation, downregulated the expression of NOX2, as well as significantly reduced myocardial infarction area and improved biochemical indicators and cardiac pathological changes, thus exerting cardiac protective effects (50). Metabolomics analysis showed that schizandrol A could also regulate myocardial injury related indicators such as glycine, serine and threonine metabolism, as well as lysine biosynthesis under acute myocardial ischemic pathological conditions (50). Furthermore, Schizandrol A might also play a role in cardioprotective effects by improving oxidative stress damage (51).

Schisandrin A, B and C isolated from *S. chinensis* extracts process a variety of nutritional and pharmacological properties. Schisandrin A exhibits significant therapeutic effects in diverse inflammatory diseases through distinct signaling pathways. In human colon cancer HT-29 cells, schisandrin A inhibited the production of intracellular reactive oxygen species (ROS) and nitrogen oxidative species induced by mycotoxin deoxynivalenol (DON), and alleviated chronic intestinal inflammatory diseases (52). Schisandrin A protected intestinal epithelial cells from cytotoxicity induced by mycophenolic acid and oxidative damage caused by increasing ROS expression, playing its antioxidant activity (53). Therefore, schisandrin A may serve as a protective against intestinal injury caused by DON and mycophenolic acid. Schisandrin A inhibited MAPK and NF-κB signaling pathways to reduce inflammation and cartilage degradation induced by IL-1β, thus playing a therapeutic role in osteoarthritis (54). Moreover, Schisandrin A may also initiate autophagy (a process in which the body activates antioxidant mechanisms to balance oxidative stress) by inhibiting the mTOR pathway and activating the adenosine monophosphate-activated protein kinase (AMPK)-unc-51 like kinase 1 (ULK1) signaling pathway, playing a protective role in lipopolysaccharide induced mouse mastitis model (55). In addition, schisandrin A may enhance the proliferation and differentiation of neural progenitor cells through cell division control protein 42, regulating cytoskeleton rearrangement and cell polarization, and improve the sequelae of ischemic brain injury (56).

Schisandrin B demonstrates substantially effects in ameliorating metabolic related diseases and repairing nervous system, primarily attributed to its antioxidant and anti-inflammatory functions. Non alcoholic fatty liver disease (NAFLD), a metabolic syndrome, is an increasingly serious public health problem affecting the world (57). A study found that schisandrin B activated autophagy by regulating the AMPK/mTOR pathway, promoting lipid clearance, inhibiting hepatic steosis, and increasing fatty acid oxidation, thus playing a role in preventing or treating NAFLD (58). Nevertheless, these preventive and therapeutic effects only observed at low doses of schisandrin B (50–200 mg/kg/day). A study found that high-dose schisandrin B lead to an increase in cholesterol and triglyceride levels in mice after a single injection (59). Therefore, it is necessary for researchers to actively explore and establish the optimal dose of schisandrin B treatment to ensure maximum efficacy and safety for human. Additionally, schisandrin B played a significant role in antioxidation, inhibiting cancer cell cycle arrest induced by cyclin D1, neuroprotection, and improvement of myocardial ischemia (60). In the cyclosporine A induced nephrotoxicity model of the human proximal tubular epithelial cell line, schisandrin B could reduce the cytotoxicity caused by oxidative stress during the application of immunosuppressants by reducing the release level of intracellular ROS and lactate dehydrogenase and increasing the level of mitochondrial membrane potential and glutathione (GSH) (61). Nuclear factor erythroid 2-related factor 2 (Nrf2) -mediated antioxidant response pathway might be the main cellular defense mechanism of antioxidant stress cytotoxicity (62). Researchers observed the behaviors of mice with and without oral administration of schisandrin B in a forced swimming mice model. The results indicated that schisandrin B increased the level of Nrf2 and decreased the level of Kelch-like ECH-related protein 1, an endogenous inhibitor of Nrf2. Additionally, schisandrin B significantly increased the expression of antioxidant molecules, including superoxide dismutase (SOD) and GSH (63). In this way, oxidative stress injury is effectively reduced and the symptoms of anxiety-like behavior induced by acute stress are alleviated, which suggested that schisandrin B may serve as a potential drug for treatment of anxiety disorders related to oxidative stress (63). The alcoholic liver disease is primarily caused by ROS. Alcohol exposure results in the production of ROS, such as superoxide, hydroxyl radical and hydrogen peroxide, and the antioxidant defense system in liver is inhibited, thereby leading to liver oxidative stress (64). Nagappen et al. found that schisandrin B (syn. Gomisin N) downregulated cytochrome P450 2E1 expression, and upregulated antioxidant gene expression, inhibited ROS production, reduced inflammatory gene expression, and reduced ethanol-induced oxidative stress, thereby exerting partial therapeutic effects on alcoholic liver disease by using mice models of chronic alcoholism and HepG2 cells treated with ethanol *in vitro* (65). JiangMeiLingJiaoNang, a commonly used liver protective drug in clinical practice, is composed primarily of schisandrin A and schisandrin B as its main components. It has demonstrated to significantly reduce the levels of Alanine transaminase, indicating its efficacy in liver protection. Li et al. found that schisandrin B elevated the level of γ-aminobutyric acid, and significantly decreased the level of glutamate in the peripheral blood of mice and in the cerebral cortex, hippocampus and hypothalamus of rats, which increased the GABA/Glu ratio, thus exerting sedative and hypnotic effects (66). Research has shown that schisandrin A and schisandrin B induce proliferation, survival, differentiation, and neurogenesis of mouse neuroectodermal neural stem cells (67). Additionally, schisandrin B treatment enhanced the expression of the neurosphere-specific adhesion molecule Cdh2 as well as Wnt pathway-related genes, including MMP9, Cyclin D1, and β-catenin, and improved nervous system development (67). Further, the oxidative stress-induced decrease in testosterone biosynthesis related-genes by using both schisandrin B (syn. Gomisin N) and schisandrol A, which are a potential therapeutic agent for treating male hypogonadism (68).

Studies have shown that excessive activation of lipolysis through autoreflexes increases the level of free fatty acids in patients with severe obesity. The release of free fatty acids from peripheral tissues in circulating may lead to obesity-related complications, including inflammation, type 2 diabetes and cancer (69). Therefore, actively controlling excessive steatolysis in obese patients is closely related to their prognosis. It has been shown that schisandrin C increases AMPK phosphorylation levels in a dose-dependent manner, which resulted in a reduciton in the protein levels of major adipogenic transcription factors (peroxisome proliferator-activated receptor γ and CCAAT/enhancer-binding protein-α) associated with lipogenesis (69). Moreover, it repressed pancrelipase activity related to fat decomposition, thereby reducing lipid accumulation (69). Shisandrin C inhibited MAPK pathway activity in C2C12 skeletal muscle cells, increased antioxidant activity and reduced ROS release, decreasing inflammatory factor levels in these cells by regulating NF-κB and Nrf2 translocation to the nucleus. Furthermore, it participates in antioxidant mechanisms by enhancing autophagy and mitochondrial biogenesis (70). In addition, schisandrin C has a significant antiviral activity. Researchers found that schisandrin C activated cyclic GMP-AMP synthase-stimulator of interferon genes pathway and increased the production of Interferon β and the expression of interferon-stimulated genes (IFIT1, ISG15, and CXCL10) to inhibit HBV replication (71). *Propionibacterium acnes* is a key pathogenic bacterium leading to acne inflammation (72). Schisandrin A, B and C effectively suppressed IL-1β secretion and pyroptosis by inhibiting NOD-like receptors family pyrin domain-containing 3 (NLRP3) inflammasome activation in human monocytes (THP-1 cells) infected with *Propionibacterium acnes*. The three lignans are a potential treatment for *Propionibacterium acnes* related infections, and the efficacy of each lignan is as follows: schisandrin C > schisandrin B > schisandrin A (73).

The development of the economy has led to an increase in social pressure, and chronic fatigue may be one of the most significant factors affecting human health in the future. Lin et al. discovered that schisantherin A, a compound found in *S. chinensis*, boosted antioxidant and anti-apoptotic activity in mice with chronic fatigue (74). This protected their brains from oxidative stress, resulting in improved learning and memory with chronic fatigue mice. Schisantherin A achieved this by decreasing the levels of certain proteins (such as Kelchlike ECH-associated protein 1, Bax, and caspase3) that cause cell death, while increasing the levels of others (such as Nrf2, heme oxygenase1 (HO-1), and Bcl2) (74). Overall, these findings suggest that schisantherin A may be a promising candidate for treating oxidative stress-related cognitive impairments. It has been found that oxidative stress negatively impacts mitochondrial biogenesis, and increased HO-1 expression can upregulate peroxisome proliferator-activated receptor-gamma coactivator 1 alpha and promote mitochondrial biogenesis (70, 75). During the oxidative stress induced by high glucose levels in diabetes, gomisin A could promote osteoblast differentiation by modulating the expression of HO-1, antioxidant enzymes, and osteoblast differentiation molecules, as well as the maintenance of mitochondrial homeostasis. Gomisin A is expected to be a potential therapeutic agent to prevent osteoporosis caused by diabetes (75). In addition, gomisin A can reduce the level of ROS in ovarian cancer cells and inhibit cancer cell proliferation by downregulating the expressions of cyclin-dependent kinase 4 and cyclin B1 (8). Gomisin A can also reduce the levels and activities of matrix metalloproteinase (MMP)-2 and MMP-9, and improve the lung metastasis of colorectal cancer cells by reducing the cell survival and metastasis ability of colorectal cancer cells (76). In the paracetamol induced acute hepatotoxicity model of mice, schisandrol B (syn. Gomisin A) significantly attenuated the increases in alanine aminotransferase and aspartate aminotransferase activity, and prevented the depletion of mitochondrial glutathione in a dose-dependent manner (77). Meanwhile, schisandrol B inhibited the biological activation of paracetamol mediated by cytochrome P450, and reduced paracetamol-induced p53 and p21 activation, as well as increased the expression of liver regeneration and anti-apoptotic related proteins such as cyclin D1, proliferating cell nuclear antigen, and B cell lymphoma/lewkmia-2, thereby protecting the liver from liver injury (77).

#### 2.2.2. Novel mass spectrometry technologies and extraction methods to analyze lignans of *Schisandra chinensis*

*S. chinensis* fermented beverage is one of the popular dietary supplements. Park et al. detected the effects of the fermentation process, fermentation time, and fermentation materials on the lignan contents in *S. chinensis* beverage by MS (6). The results shown that the total content of schisandrol A, schisandrol B, tigloylgomisin H, angeloylgomisin H, schisandrin A, schisandrin B, and schisandrin C in the seeds, flowers, leaves, pulp, and stem of *S. chinensis* decreased sequentially (6). And the total lignan content in *S. chinensis* beverage fermented with white sugar for 12 months increased by 2.6 times, while fruit wine made by soaking fruits in alcohol had a higher total lignan content, which may be related to the fact that alcohol itself is a solvent for extracting the effective components of *S. chinensis* (6). Meanwhile, compared with unprocessed *S. chinensis*, wine processed *S. chinensis* has a more significant role in regulating gut microbiota derivatives related to GPR81 receptor-mediated lipid metabolism pathways, thereby improving anxiety and depression-like behaviors in rats (78). Taking into account the complexity of the *S. chinensis* components, further exploration of the potential value of *S. chinensis* beverages processed with alcohol is necessary. Separation and purification methods are essential for obtaining high-purity extracts from *S. chinensis* during the process of obtaining, identifying, and utilizing *S. chinensis* components. Supercritical fluid extraction with the characteristics of being green, mild, and selective, can effectively remove residual solvents from extracts (79). The lignans from different parts of *S. chinensis* plant were extracted by using supercritical fluid extraction technology with CO_2_ as the supercritical solvent and ethanol as the co-solvent (79). Finally, 36 compounds were isolated from leaves, and 43 compounds were isolated from wooden stems, while 36 compounds were isolated from rhizomes and roots. Then the samples were accurately analyzed by HPLC-SPD-ESI-MS/MS. High-precision mass spectrometric data were recorded on an ion trap equipped with an ESI source in the mode of negative ions by a three-stage ion separation mode (79). An analysis of literature revealed that 26 bioactive substances are classified as lignans, namely schisandrin C, gomisin M1, gomisin L2, gomisin M2, gomisin J, pregomisin, schisandrin B, schisanhenol, gomisin O, erigomisin O, schisandrin A, demethylated metabolites of schisandrol A, schisandrol A, 7, 8-Dihydroxy-schisandrin, tigloylgomisin O, angeloylsogomisin O, angeloygomisin H, micrantherin A, gomisin E, schisantherin D, benzoylgomisin O, benzoylgomisin H, gomisin D, gomisin G, schisantherin A and benzoylgomisin Q (2). There has been evidence that gomisin L1 (gomisin M1) has strong anti-HIV properties (80). Using *siRNA* to knockdown NADPH oxidase (NOX), it was demonstrated that gomisin L1 increased intracellular ROS levels and promoted apoptosis in ovarian cancer cells through NOX (81). In addition, α-Iso-cubebene (ICB), another compound of dibenzocyclooctadiene lignans in *S. chinensis*, could inhibit high mobility group box 1 (HMGB1)-induced monocyte–macrophage differentiation by reducing the level of aberrantly expressed ROS in monocytes, which was important in the treatment of vascular inflammation and subsequent endothelial proliferation associated with vascular injury (82). Piao et al. developed a magnetic separation method based on polyethylenimine-modified magnetic nanoparticles (PEI-MNPs). The method formed a strong cation-π interaction with the benzene rings of lignans through the─NH_3_^+^ groups on the PEI-MNPs surface, and the methoxy on the benzene ring enhanced the negative electron cloud density, and strengthened the cation-π interactions and electrostatic adsorption ability (83). This method allowed for the rapid and effective isolation and purification of lignans, followed by the characterization of magnetic nanoparticles by transmission electron microscopy, vibrational sample magnetometer, Fourier transform infrared spectroscopy and X-ray diffraction (83). Finally, in addition to the 26 lignan components mentioned above, 13 other components including schisandrol B, tigloylgomisin H, angeloylgomisin Q, gomisin F, gomisin K1, gomisin K2, schisantherin B, tigloylgomisin P, schisantherin C, epigomisin O, benzoylisogomisin O, angeloylisogomisin O, angeloylgomisin O have been detected (83). An integrated method based on UPLC-high resolution (HR) MS coupled with characteristic fragment filtering and online database query was used for *S. chinensis* composition analysis (84). A total of 94 compounds were primarily or definitively characterized, including 58 lignans, 15 triterpenoids and 21 other compounds. After analytical comparison, 16 of these compounds did not match with those in the established *S. chinensis* chemical composition database and were therefore identified as potential new compounds. In the study, lignans were found to be abundant in *S. chinensis*, and the study recommended using LC–MS to identify potential new components of the plant (84). Using advanced HR-MS techniques, Yang et al. isolated and identified a new lignan (7R,7′R,8R,8′R)-8-hydroxypinoresinol 8-O-β-d-glucopyranoside, named schilignan F, from the rattan stems of *S. chinensis* (85). Liu et al. isolated a new lignan (schisandroside E) and a new terpenoid (schisandenoid A) from *S. chinensis* leaves by using nuclear magnetic resonance (NMR), MS, infrared spectroscopy, ultraviolet spectroscopy and circular dichroism (CD) (86). Ying et al. structurally analyzed the compounds extracted from the *S. sphenanthera* stem by NMR, HR-ESI-MS and CD, and identified a new dibenzocyclooctadiene lignan, sphaerandrin A (87). However, the functions and value of these new components remain to be further investigated.

The contents of lignan in *S. chinensis* is also an important indicator for its quality. The 2020 edition of The *Chinese Pharmacopeia* specifies that the content of schisandrol B should not be less than 0.40%, while the *US Pharmacopeia* specifies that the percentage of total content of schisandrin, schisandrol B, deoxyschisandrin and γ-schisandrin should not be less than 0.95% of the lignan content based on HPLC (88). Schisandrol A, schisandrol B, angeloylgomisin H, gomisin G, schisantherin A, schisanhenol and schisandrin A were quantitatively analyzed in 43 batches of *S. chinensis* samples collected from different locations using HPLC-DAD-MS. The results suggested that schisandrol A, schisandrol B, schisandrin B, and schisandrin C could be used as chemical markers in the evaluation of high quality *S. chinensis* (89). Meanwhile, researchers found that the total amount of nine lignans in *S. chinensis* collected from Heilongjiang and Liaoning provinces was significantly higher than that from other regions, indicating that the geographic environment in Northeast China is more suitable for the growth of *S. chinensis* (89). A study in 2022 analyzed 14 preparations of *S. chinensis* by HPLC fingerprinting combined with multiple chemometric methods and discovered that schisandrin A, besides the four components mentioned above, could also be used as a comprehensive marker for quality control of *S. chinensis* (90).

Chen et al. identified 15 components simultaneously in wild and cultivated *S. chinensis* using ultra-fast performance liquid chromatography coupled with triple quadrupole linear ion trap mass spectrometry (UFLC-QTRAP-MS/MS), including 11 lignans (schisandrin, gomisin D, gomisin J, schisandrol B, angeloylgomisin H, schizantherin B, schisanhenol, deoxyschizandrin, γ-schisandrin, schizandrin C, and schisantherin), and four organic acids (quinic acid, D(−)-tartaric acid, L-(−)-malic acid, and protocatechuic acid) (88). The results showed that the content of effective components in cultivated *S. chinensis* was lower than that in the wild type, and the wild type *S. chinensis* has a better quality.

### 2.3. Triterpenoids

Triterpenoids are found in a variety of parts of *S. chinensis*, including fruit, leaves, vine stems and roots, and are second only to lignans in terms of content (19). During June 2014 to November 2021, 211 new triterpenoids were isolated and identified from 8 species of plants in the Schisandraceae family (91). The new triterpenoids can be classified as lanostanes, cycloartanes, dammaranes and ursanes and schinortriterpenoids. These compounds display a wide range of pharmacological activities, including antiviral, antitumor, anti-inflammatory, hepatoprotective, immunosuppressive activity and neuroprotective functions (91). Song et al. (92) isolated a new highly oxidized triterpenoid schinchinenlactone D from *S. chinensis* roots along with three known triterpenoid compounds propinic lactone A, schisanbilactone A and kadsudilactone C using HR-ESI-MS. All the three compounds exhibited anti-inflammatory activity with exception of kadsudilactone C. Moreover, schisanbilactone A showed the strongest anti-inflammatory effects (92). Using UHPLC-Q-TOF-MS with positive or negative ions produced by different types of compounds, Yang et al. identified target compounds using their characteristic fragments (93). There were a total of nine triterpenoids were identified in *S. chinensis*. They included two cycloartane-type triterpenoids, three lanostane-type triterpenoids, three schisanra-type triterpenoids and one other terpenoids (93). Using 1D and 2D NMR spectroscopy, single crystal X-ray diffraction, and HRMS, Qiu et al. identified three new triterpenoids (schisanlactone I, schinalactone D, schisanlactone J) from *S. chinensis* and four known triterpenoids (kadsuphilactone B, schisanlactone C, schisphendilactone B, and schinchinenlactone A). Despite testing these seven compounds against the HepG2 cell line, none of them showed any cytotoxic effect (94). In addition, several novel triterpenoid compounds were also isolated from *S. sphenanthera* by mass spectrometry. Liu et al. (95) isolated three novel (schisphenthin A, B and C) and seven known triterpenoids from the *S. sphenanthera* fruits using HR-ESI-MS and other spectroscopic analysis techniques. Schisphenthin A and schisphenthin C exhibited moderate antiproliferative effects against HepG2 cells. Using the same MS method, Liang et al. isolated two new triterpenoids (schisphendilactone A and B) and three known triterpenoids (nigranoic acid, kadsuric acid and lancifoic acid A) from the stem of *S. sphenanthera*. Schisphendilactone B had the effect of inhibiting the activity of several cancer cell lines (HL-60, SMMC-7721, A-549, MCF-7, and SW-480), and lancifoic acid A exhibited anti-HIV-1 activity (96).

### 2.4. Organic acids

Organic acids are crucial for preserving the nutritional value and flavor of food and they are widely used as food additives for their preservative, acidity-regulating, and antioxidant properties (97). *S. chinensis* contains approximately 18% organic acids, mainly citric acid, which is the primary source of its sour taste (98). Using HPLC and other techniques, researchers dscovered that fresh and mature *S. chinensis* fruit contains 3.26% ± 0.06% citric acid, 1.13% ± 0.04% malic acid, and 0.53% ± 0.01% shikimic acid (99). In a second study, UHPLC-Q-TOF-MS was utilized to identify citric acid, 6-methyl citrate, and dimethyl citrate from *S. chinensis* (93). Organic acids are commonly used in the food industry to prevent the enzymatic browning of fruits and vegetables induced by polyphenol oxidase (100). Enzymatic browning can alter the nutritional characteristics and appearance of fruits and vegetables. As a result, organic acids play crucial role in acting as natural food preservatives (100). The presence of organic acids in food acts an natural antibacterial agent. A study has shown that organic acids may exert bacterial inhibitory effects by disrupting the bacterial internal balance and inhibiting enzyme activity (101). The orle of organic acids in nutrition and health can be explained by their ability to regulate energy and metabolism, strengthen the human immune system, and protect the heart (97). In the clinical setting, *S. chinensis* is commonly steamed with vinegar to enhance its acidic and astringent properties according Chinese medicine theory. Yin et al. investigated the effects of vinegar processing on its organic acid composition (102). The gas chromatography was conducted to identify 39 organic acid compounds isolated from *S. chinensis* through a methylation reaction. In comparison to *S. chinensis* that has not been processed, processed *S. chinensis* with vinegar has a significantly high amount of levulinic acid. An experimental study has shown that levulinic acid inhibits the contractile force of isolated intestines and inhibits on the excessive hyperactivity of small intestinal propulsion (102).

### 2.5. Polysaccharides

*S. chinensis* fruits contain approximately 1.5% sugar, including polysaccharides and monosaccharides (103). Polysaccharides are composed of more than 10 monosaccharides linked by glycosidic bonds (104). A HPLC analysis of *S. chinensis* polysaccharides revealed 64% glucose, 17.7% galacturonic acid, 9.0% galactose, 3.9% Rhamnose, 3.5% mannose, 1.2% xylose, and 0.8% arabinose as monosaccharides (105). Likewise, the structure and content of polysaccharides were also different between *S. chinensis* and *S. sphenanthera*. Previous studies have shown that the type and content of polysaccharides isolated from northern *S. chinensis* were much higher than those from *S. sphenanthera* (1). The polysaccharides in *S. chinensis* and *S. sphenanthera* were analyzed by gas chromatography coupled with Fourier transform infrared spectroscopy, and the result revealed that with respect to free radical scavenging ability, protective effects on biomolecules, cellular antioxidant activity, *S. sphenanthera* had stronger effects than *S. chinensis*, which was largely due to its high levels of protein and uronic acid; *S. chinensis* had stronger cell viability, neutral red phagocytosis, NO production, and acid phosphatase activity compared with *S. sphenanthera*, owing to its higher mannose, galactose, arabinose, and glucose contents, and these functions were closely related to enhancement of the body’s immunity (106). Li et al. (7) extracted and purified *S. chinensis* fruit with ethanol, and the structural characteristics and physicochemical properties of polysaccharides were analyzed by using the technology of hydrophilic interaction liquid chromatography-negative electrospray-mass spectrometry (HILIC-ESI-MS) combined with microwave assisted mild acid (MAMA) depolymerization, which enabled the rapid identification of *S. chinensis* and *S. sphenanthera* by making polysaccharides as quality evaluation indicators of Chinese medicinals. In addition, the polysaccharide content of *S. chinensis* varied across different regions. Wu et al. used near-infrared spectroscopy combined with chemometrics to determine the total polysaccharide content of *S. chinensis* grown in the provinces of Liaoning, Jilin, and Heilongjiang. Results revealed that samples from Heilongjiang province had the highest content of polysaccharides followed by those from Jilin province (107).

There are a number of therapeutic effects associated with *S. chinensis*. As an example, polysaccharides were found to significantly lower serum transaminase levels in mice suffering from immunological liver injury induced by concanavalin A, inhibit the release of a large number of inflammatory factors, and alleviate the effects of oxidative stress response by regulating Nrf2/antioxidant response element and TLR4/NF-κB signaling pathway (108). Additionally, the polysaccharides isolated from *S. chinensis* stem also exerted hepatoprotective effects by increasing the expression of UDP-glucose pyrophosphorylase and UDP-glucose 6-dehydrogenase, while decreasing the expression of acetyl coenzyme A carboxylase and fatty acid synthase in the liver of rats with NAFLD, thereby alleviating the development of NAFLD (109). *S. chinensis* polysaccharides also reduced the expression of pro-inflammatory cytokines and the activation of glial cells in the hippocampus, improving cognitive performance and histopathological changes in mice model of Alzheimer’s disease (110). The drug of cyclophosphamide is an anti-tumor medication, but it has a number of immunosuppressive side effects which may complicate treatment. Consequently, it is essential for patients receiving cyclophosphamide treatment to take food supplements with immune regulatory functions at the same time. Water extraction and ethanol precipitation methods were used to obtain *S. chinensis* polysaccharides from dried fruits of *S. chinensis*, and the monosaccharide composition of *S. chinensis* polysaccharides was determined by HPLC. ICR mice were continuously gavaged with the polysaccharides for 21 days, and peritoneal injection of cyclophosphamide for 5 days starting from the 17th day after administration (111). The experimental results showed that *S. chinensis* polysaccharides significantly inhibited cyclophosphamide-induced lymphocyte apoptosis and thymus and spleen damage, and effectively prevented cyclophosphamide-induced cellular immunity, humoral immunity and nonspecific immune damage. Therefore, *S. chinensis* polysaccharides may be used as an adjuvant immune enhancer for prevention of immune deficiency (111).

In recent years, researchers have continuously isolated and identified a variety of new polysaccharides from *S. chinensis* and *S. sphenanthera*. Niu et al. (112) isolated and purified a water-soluble polysaccharide (SSPW1) with an average molecular weight of 191.18 kDa from the aqueous extract of *S. sphenanthera*. The component of SSPW1 was analyzed by gas chromatography, which contained 48.92% neutral sugars, 5.56% proteins and 42.83% glyoxylates. The monosaccharide component analysis suggested that SSPW1 was composed of rhamnose, arabinose, mannose, galactose, and glucose. Experiments *in vitro* and *vivo* showed that SSPW1 had antioxidant effects and could increase body weight and improve glucose tolerance in diabetic rats (112). Antibiotic associated diarrhea is caused by the toxic side effects of antibiotics themselves (113). Qi et al. (114) investigated the effect of water-soluble polysaccharides of *S. chinensis* on antibiotic-associated diarrhea in rats. The researchers extracted polysaccharides from the dried fruits of *S. chinensis*, and the extracted polysaccharides were used in a rat model with antibiotic-associated diarrhea, and the monosaccharide composition of water-soluble polysaccharides was analyzed by using HPLC. The results showed that water-soluble polysaccharides might be a potential natural product for treatment of antibiotic-associated diarrhea (114). Zhao et al. isolated and identified a water-soluble low molecular weight polysaccharide SCPP11 from *S. chinensis* with a molecular weight of 3.4 kDa, and the structural characteristics of SCPP11 were comprehensively analyzed by using gas chromatography-MS (GC–MS), NMR, high-performance size exclusion chromatography-angle laser light scatteringrefractive index detector (HPSEC-MALL-RI), CD, atomic force microscope (AFM) and transmission electron microscope (TEM) (115). The research found that SCPP11 elevated the thymic index and serum levels of IL-2 and TNF-a in mice with hepatocellular carcinoma, and significantly increased the phagocytic activity of macrophages *in vitro*, thus improving the immune response and exerting antitumor effects. SCPP11 might be used as a potential anticancer adjuvant in health foods and therapeutic drugs (116).

Zhong et al. isolated and identified a kind of polysaccharide-1 (SCFP-1) from *S. chinensis* fruits, with a molecular weight of 31.8 kDa, by gel permeation chromatography (GPC) and GC–MS, which mainly composed of 66.5% glucose and 29.4% arabinose (117). Applying polysaccharide-1 to a cough guinea pig model, it was found that polysaccharide-1 could reduce the cough frequency and airway inflammation of guinea pig models caused by cigarette smoke-induced cough hypersensitivity, and could significantly alleviate acute cough caused by citric acid. It indicated that polysaccharide-1 was one of the cough suppressant components of *S. chinensis*, which provided an important compositional support for the development of new cough suppressant drugs (117). Xu et al. characterized the polysaccharide components of *S. chinensis* extract and obtained a polysaccharide SCP2-1, with a molecular weight of 5.388 kDa, consisting of glucose and galactose in a molar ratio of 8.78:1.23 by using HPLC-GC–MS, NMR, and Fourier transform infrared spectroscopy (118). In the mice models of lipopolysaccharide-induced cognitive dysfunction, researchers found that SCP2-1 treatment reduced the sustained release of pro-inflammatory cytokines such as TNF-α and IL-1β and upregulated the level of anti-inflammatory cytokines such as IL-4 and IL-10 by inhibiting nuclear translocation of NF-κB and preventing overactivation of the P38 MAPK pathway, thereby reducing excessive inflammatory responses (118). Meanwhile, SCP2-1 reduced the expression level of NLRP3, M-caspases-1, and reduced the excessive deposition of β-amyloid (Aβ) associated with neuronal degeneration, thereby exerting a neuroprotective effect (118). In 2023, Fu et al. used a metabolomics method for serum and urine based on UPLC-Q-TOF-MS to isolate and purify a homogeneous polysaccharide SCP2 from *S. chinensis* polysaccharide, then investigated the therapeutic effects and potential mechanisms for Alzheimer’s disease in rats (119). The results showed that SCP2 significantly reversed the metabolic profile disorder in Alzheimer’s disease rats and played an important role in ameliorating Aβ_25-35_-induced cognitive dysfunction, attenuating oxidative damage, and reducing Aβ deposition in the hippocampus, which may provide new insight into the potential mechanisms of SCP2 treatment of AD (119). Zhao et al. isolated a new polysaccharide SCPP22, with a molecular weight of 143 ± 0.13 kDa from *S. chinensis* fruit by HPLC, GC–MS, and NMR. SCPP22 is mainly composed of glucose and galactose (120). Researchers further investigated the function of this polysaccharide and found that SCPP22 may reverse polychlorinated biphenyl (PCB126)-induced immunosuppression by regulating the expression of apoptosis-related proteins, and increase the level of SOD activity and reduce malondialdehyde (MDA) in the spleen and thymus, thus ameliorating oxidative damage in immune organs (120). Polychlorinated biphenyls (PCBs) are ubiquitous environmental contaminants, which can attack the immune and nervous systems after ingesting by people (121). Therefore, SCPP22 may be used as a nutritional intervention component in functional foods to reduce the adverse effects of PCB contamination.

Recently, ultra-high performance supercritical fluid chromatography coupled with DAD was used for the first time to analyze the whole monosaccharide components of *S. chinensis* fruit polysaccharides. This study provided a new alternative method for determining the monosaccharide components of natural polysaccharides (122). Liu et al. (123) proposed a sample preparation method based on microwave assisted free radical degradation (MFRD) of plant polysaccharides to efficient degradation of *S. chinensis* and *S. sphenanthera* polysaccharides. HILIC-ESI-Q-TOF-MS and quadrupole-orbitrap-ion trap-tandem MS were used to characterize a series of oligosaccharides and small molecular weight polysaccharides after degradation. By using a high-performance anionexchange chromatography with pulsed amperometric detection (HPAEC-PAD) combined with MFRD, a low-polymerization compositional fingerprinting was successfully constructed for *S. chinensis* and *S. sphenanthera* in order to improve the quality assessment of *S. chinensis* (123). A novel, recyclable and green temperature-responsive deep eutectic solvent was applied to the simultaneous extraction and separation of different polar active phytochemicals from *S. chinensis*. Under the optimal parameters, the extraction yields of lignans (water, methanol and 70% ethanol) and polysaccharides (water extraction) are 1.62–1.17 times and 1.39 times higher than those of traditional solvents, respectively. Consequently, the application of temperature-responsive deep eutectic solvent could improve the extraction efficiency and enable cost savings (124). In addition, Du et al. have synthesized *S.chinensis* polysaccharide-conjugated selenium nanoparticles (SCP-Se NPs) for the first time, thereby enhancing the activity of polysaccharides, and compared with *S. chinensis*, SCP-Se NPs were more effective in alleviating diarrheal symptoms and intestinal tissue damage and reducing the level of pro-inflammatory cytokines (125). These results would further strengthen researchers to understand the functions of *S. chinensis* fruit polysaccharides and promote their potential applications in functional foods and pharmacological fields. The application of new mass spectrometry detection technologies combined with advanced extraction and analysis methods has greatly improved the discovery of novel polysaccharide components in *S. chinensis*. However, there is still a need for systematic and comprehensive investigations to fully understand the nutritional functions of these components.

## 3. Conclusions and perspectives

**Figure 1:**
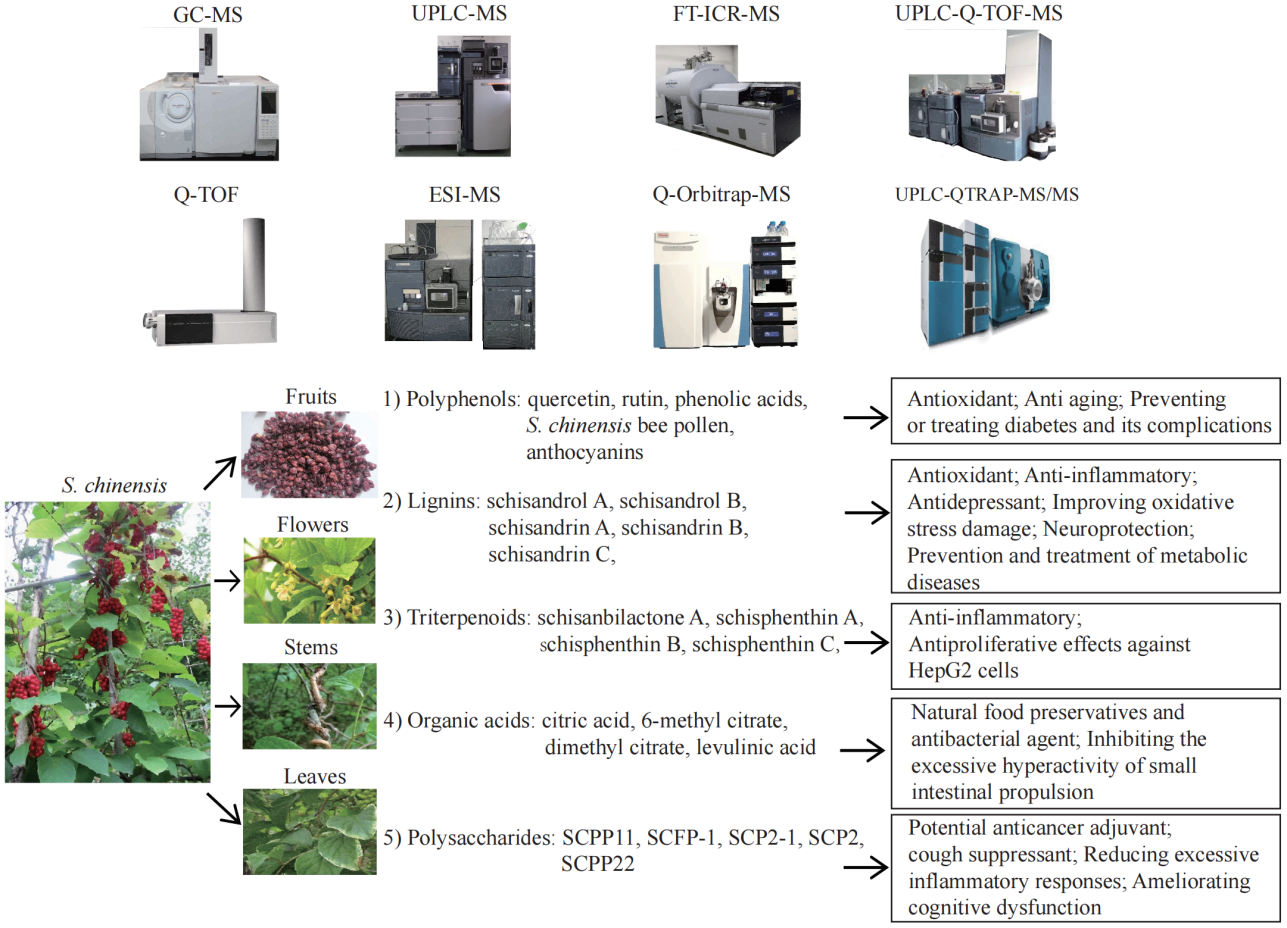
The mass spectrometry technologies and nutritional effects of *Schisandra chinensis* components. ESI-MS, electrospray ionization mass spectrometry; FT-ICR-MS, fourier transform ion cyclotron resonance MS;GC–MS, gas chromatography MS; Q-Orbitrap-MS, quadrupole Orbitrap MS; Q-TOF-MS, ultra-high performance liquid chromatography quadrupole time of flight MS; UPLC-MS, ultra-high performance liquid chromatography MS; UPLC-Q-TOF-MS, ultra-high performance liquid chromatography Q-TOF-MS; UPLC-QTRAP-MS/MS, UPLC coupled with triple quadrupole-linear ion trap MS.

*S. chinensis* contains a variety of bioactive ingredients including rich amount of natural plant nutrients, that provide a variety of nutritional and therapeutic benefits. *S. chinensis* primarily exerts its effects on antioxidation. Antioxidative process involves the removal of excess ROS in the body in order to prevent oxidative stress from damaging cells and secondary apoptosis induced by oxidative stress (126, 127). ROS are closely associated with aging and the progression of various diseases, including cancer (8, 26, 81, 126). The antioxidant is therefore one of the most important research and development directions in the health category, as well as one of the most important functional demands on the market. Polyphenols, also known as “the seventh category of nutrients,” are composed primarily of flavonoids, phenolic acids, and anthocyanins, which are responsible for the antioxidant function of *S. chinensis*. Polyphenols are often used as a dietary ingredient and nutritional supplement in the manufacture of foods that are considered as functional foods. Polyphenols are abundant in *S. chinensis* leaves, thus fully extracting polyphenol compounds from *S. chinensis* leaves will increase the beneficial for improving the utilization value of the plant. In addition to polyphenols, various components of Schisandra lignans, such as schisandrol A, schisandrin A, B and C, gomisin D, schisandrol B, schisanterin A, schisanterin C, kadsuranin, and kadlongilactone F, and gomisin L1, as well as organic acids and polysaccharides, all exhibit strong antioxidant effects and are closely associated with anti-inflammatory effects. Due to the above-mentioned efficacy, *S. chinensis* represents an excellent antioxidant with broad application potential. The bioactive nutrients in *S. chinensis* can help prevent, manage, and treat neurological conditions. A major role of *S. chinensis* is to function as antidepressant, an anti-anxiety, sedative and hypnotic agent, to improve learning and memory, and to improve cognitive dysfunction by regulating the relevant signal pathways in the hippocampus. A variety of active ingredients, including schisandrin A, B and C, schisanterin A, gomisin A, triterpenoids, and water-soluble polysaccharide (SSPW1), are also capable of improving a variety of metabolic diseases, including NAFLD, alcoholic liver disease, obesity, diabetes and related complications, through their antioxidant, anti-inflammatory, and maintaining mitochondrial homeostasis. In addition, organic acids, one of the *S. chinensis* extracts, are the natural food preservative that maintain the nutrients and appearance of fruits and vegetables. As natural antibacterial agents, organic acids, quercetin, schisandrin A, B and C, mannose, galactose, arabinose and glucose are also beneficial to boost immunity. While cyanidin 3-Rutinoside has the potential as a therapeutic agent for allergic diseases. Figure 1 was created to provide a clear and direct understanding of the topic discussed in this review.

Applying new extraction methods and techniques may improve the detection rate and purity of *S. chinensis* components; on the other hand, combination with new MS technologies may facilitate the identification of active ingredients and further functional analysis. The components and effects of *S. chinensis* detected by MS in recent years are summarized in Table 2. In current research, more attention is focused on the lignans and polysaccharides of *S. chinensis* extracts than on new components and new effects of *S. chinensis* triterpenoids, polyphenols, and organic acids. At present, dietary supplements primarily containing *S. chinensis* on the market focus on enhancing immunity, improving sleep, protecting the liver, and preventing fatigue. Developing new formulations and functionalities for *S. chinensis* dietary supplements will be a priority in future research. It is necessary to fully utilize mass spectrometry technology to explore the resources of *S. chinensis* components, thus benefiting human health.

**Table 2:** The components and effects of *Schisandra chinensis* detected by MS.

Types	MS methods	Components	Effects	References
Polyphenols	HPLC-ESI-MS	Cya-3-*O*-xylrut	Antioxidant	(37)
	GC–MS; ESI-TQMS	Cyanidin 3-Rutinoside	Inhibiting allergic inflammation	(39)
Lignans	HPLC-ESI-TOF-MS	Gomisin D	scavenging ABTS+ radicals	(46)
	SFC-DAD	schisandrin	Regulating the gut microbiota; Antidepressant	(48)
	UPLC-Q-TOF-MS	schisandrol A	Inhibiting pulmonary fibrosis; Improving symptoms of acute myocardial ischemia	(49, 50)
	UHPLC	Schisandrin B	Inhibiting hepatic steatosis and promoted fatty acid oxidation and improving NAFLD	(58)
	HPLC	Schisandrol A; gomisin N	promoting testosterone biosynthesis	(68)
	HPLC–MS/MS	Schisandrin C	Inhibiting the production and trypsin levels	(69)
	HPLC-ESI-MS	Schisantherin A	Improving the learning and memory abilities of chronic fatigue mice	(74)
	LC-MS/MS	schisandrol B(gomisin A)	Improving APAP-induced acute hepatotoxicity and promoting liver regeneration	(77)
	HR-EI-MS	gomisin L1	Anti-HIV	(80)
	HPLC	α-Iso-cubebene	Inhibiting vascular inflammation with subsequent intimal hyperplasia related to vascular injury	(82)
Triterpenoids	HR-ESI-MS	schinchinenlactone D, Propinic lactone A, Schisanbilactone A	Inhibiting inflammation	(92)
Organic acids	HPLC; GC-MS	Levulinic acid	Inhibiting the excessive hyperfunction of small intestinal propulsion	(102)
Polysaccharide	HPLC	WSP	Improving the gut microbiota and antibiotic-associated diarrhea	(113)
	GC–MS	SCPP11	Improving immune response and anti-tumor effects	(115)
	GPC; GC-MS	polysaccharide-1	Alleviating airway inflammation in chronic cough guinea pigs to exert antitussive effects	(116)
	HPLC-GC-MS; FTIR	SCP2-1	Improving LPS-induced cognitive dysfunction in mice and ameliorating excessive inflammatory response	(117)
	UPLC-Q-TOF-MS	SCP2	Improving cognitive dysfunction and reversing the metabolic profile disorder in Alzheimer’s disease rats	(118)
	HPLC; GC-MS	SCPP22	Reversing PCB126-induced immunosuppression	(119)

DAD, diode array detection; EI, electron ionization; ESI, electrospray ionization; FTIR, fourier transform infrared spectroscopy; GC, gas chromatography; GPC, gel permeation chromatography; HPLC, high performance liquid chromatography; HR, high-resolution; LC, liquid chromatography; MS, mass spectrometry; NAFLD, non alcoholic fatty liver disease; Q-TOF, quadrupole time of flight; SFC, supercritical fluid chromatography; TQ, triple quadrupole; UHPLC,ultra high performance liquid chromatography; UPLC, ultra performance liquid chromatography; WSP, water-soluble polysaccharides.

## Author contributions

MJ and LZ performed the literature search and wrote the manuscript. YL performed the literature search. HZ drafted the tables. XY and XO revised the manuscript. YH supervised and revised the manuscript. All authors contributed to the article and approved the submitted version.

## Funding

This work was supported by General Projects of National Natural Science Foundation of China in 2021 (82174187), 2022 Leading Talents of Science and Technology Innovation in Zhongyuan (234200510028), and Leading Talent Project of Traditional Chinese Medicine in Henan Province (No. [2021] No. 8).

## Conflict of interest

The authors declare that the research was conducted in the absence of any commercial or financial relationships that could be construed as a potential conflict of interest.

## Publisher’s note

All claims expressed in this article are solely those of the authors and do not necessarily represent those of their affiliated organizations, or those of the publisher, the editors and the reviewers. Any product that may be evaluated in this article, or claim that may be made by its manufacturer, is not guaranteed or endorsed by the publisher.

## Supplementary material

The Supplementary material for this article can be found online at: https://www.frontiersin.org/articles/10.3389/fnut.2023.1227027/full#supplementary-material
