# Supplementary material for: An analysis of the nutritional effects of Schisandra chinensis components based on mass spectrometry technology
Source: Front Nutr. 2023 Jul 25;10:1227027. doi: 10.3389/fnut.2023.1227027 (PMC10408133; doi:10.3389/fnut.2023.1227027)
Supplement: Supplementary file 1 [file Table_1.DOCX]

| Table S1. The advantages and disadvantages of utilizing different new mass spectrometry techniques for the component analysis | | | |
| --- | --- | --- | --- |
| Mass Spectrometry Techniques | Advantages | Disadvantages |  |
| GC-MS | Characterization of low-molecular weight and medium or low polarity compounds, in particular, primary metabolites such as fatty acids, carbohydrates, amino acids and all volatile compounds | Non-volatile compounds need to be derivatized prior to analysis. |  |
| LC-MS | Characterization of non-volatile compounds with higher polarity and/or molecular weights | Not suitable for characterization of low-molecular weight and medium or low polarity compounds |  |
| UHPLC | Analysis of complex samples; Higher speed, sensitivity and resolution; reduction of analysis time and solvent consumption | Higher pressures result in shorter pump life |  |
| Q-TOF-MS | Providing high-resolution spectrogram; Analysis of large molecular weight and complex samples in life science. | High equipment cost and maintenance expense |  |
| FT-ICR-MS | Excellent qualitative ability; High resolution; High sensitivity | Slower analysis speed |  |
| HR-ESI-MS | Providing accurate molecular weight and structure information at the same time; Multiple ionization modes for selection | The selection of solvents and the range of solutions available are limited; The experimental parameters or technical conditions must be selected according to the problems to be solved |  |
| Orbitrap-MS | High accuracy of analysis quality | Low resolution and sensitivity compared with FT-ICR-MS |  |
| ESI, electrospray ionization; FT, fourier transform; GC, gas chromatography; HR, high-resolution; ICR, ion cyclotron resonance; LC, liquid chromatography; MS, mass spectrometry; Q-TOF, quadrupole time of flight; TOF, time of flight; TQ, triple quadrupole; UHPLC, ultra high performance liquid chromatography; | | | |
